# Investigation of Type A Aortic Dissection Using Computational Modelling

**DOI:** 10.3390/biomedicines12091973

**Published:** 2024-09-01

**Authors:** Mohammad Al-Rawi, Djelloul Belkacemi, Eric T. A. Lim, Manar Khashram

**Affiliations:** 1 Center for Engineering and Industrial Design (CEID), Waikato Institute of Technology (Wintec), Hamilton 3240, New Zealand; 2 Faculty of Engineering, Chemical and Materials Engineering, University of Auckland, Auckland 1010, New Zealand; 3 Unité de Développement des Equipements Solaires UDES, EPST Centre de Développement des Energies Renouvelables (CDER), Bousmail, Tipaza 42415, Algeria; belkacemidjelloul@gmail.com; 4 Department of Vascular & Endovascular Surgery, Waikato Hospital, Hamilton 3204, New Zealand; eric_lta@hotmail.com (E.T.A.L.); manar.khashram@gmail.com (M.K.); 5 Department of Surgery, University of Auckland, Auckland 1023, New Zealand

**Keywords:** aortic dissection, computational modelling, wall shear stress (WSS), endothelial cell activation potential (ECAP)

## Abstract

Aortic dissection is a catastrophic failure of the endothelial wall that could lead to malperfusion or rupture. Computational modelling tools may help detect arterial damage. Technological advancements have led to more sophisticated forms of modelling being made available to low-grade computers. These devices can create 3D models with clinical data, where the clinical blood pressure waveforms’ model can be used to form boundary conditions for assessing hemodynamic parameters, modelling blood flow propagation along the aorta to predict the development of cardiovascular disease. This study presents patient-specific data for a rare case of severe Type A aortic dissection. CT scan images were taken nine months apart, consisting of the artery both before and after dissection. The results for the pre-dissection CT showed that the pressure waveform at the ascending aorta was higher, and the systolic pressure was lagging at the descending aorta. For the post-dissection analysis, we observed the same outcome; however, the amplitude for the waveform (systolic pressure) at the ascending aorta increased in the false lumen by 25% compared to the true lumen by 3%. Also, the waveform peak (systolic) was leading by 0.01 s. The hemodynamic parameter of wall shear stress (WSS) predicted the aneurysm’s existence at the ascending aorta, as well as potential aortic dissection. The high WSS contours were located at the tear location at the peak blood flow of 0.14 s, which shows the potential of this tool for earlier diagnosis of aortic dissection.

## 1. Introduction

Early diagnosis of cardiovascular diseases such as aortic dissection (AD) could save patients from complicated surgical procedures or death [1]. The contemporary incidence of AD is estimated to affect 3.13 per 100,000 persons [2]. One of the consequences of AD is the changes to the aortic geometry, such as splitting the aortic lumen into a true and false lumen, by tears in the aortic wall [3]. Therefore, clinicians use computational modelling analysis to identify advanced AD prediction risk factors [4,5,6,7]. AD can be divided into two types: Type A which involves a tear that starts at the ascending aorta, close to the heart, whereas with Type B, the tear starts distal to the left subclavian artery. Aortic dilatation and aneurysms are known risk factors for aortic dissection occurring at the ascending or descending aorta, but other underlying genetic triggered aortopathy might also play a role [8].

Hemodynamic mechanical stresses acting on the aortic wall gives rise to an intimal tear, which ultimately progresses to a dissection [9]. This dissection entails a split in the aortic wall, creating two lumens (one false and one true), with the false lumen (FL) taking most of the blood flow, which may cause serious short- and long-term complications. The natural history of AD is usually a sudden presentation without a complete understanding of the underlying mechanisms that have led to this event. However, some patients with AD can present in the chronic phase with symptoms or may have an incidental finding of a chronic dissection. One utility of computational modelling is to provide the ability for clinicians to potentially enable the knowing of the location that dissection tear would most likely occur or identify patients that might have high-risk features in the aorta that are more likely to cause an AD.

Current computational fluid dynamics (CFD) studies have presented patient-specific data relating to the different AD types. Alimohammadi et al. [9] studied a female patient, aged 54 years, presenting with cardiovascular complications such as hypertension, chest pain, and back pain who was diagnosed with a type B AD at the arch branches. The authors converted CT scan images into a 3D model showing the aorta, excluding the iliac bifurcation, simulated using Windkessel boundary conditions, and treating the artery wall as a rigid model. The blood properties were assumed as non-Newtonian (Carreau–Yasuda) using the k-omega shear stress transport (SST) model. Their results showed high wall shear stress (WSS) contours in the corresponding AD region. Furthermore, they observed high time-averaged WSS (TAWSS) and oscillatory shear index (OSI) at the branches, even though the flow rate was lower in the true lumen. Therefore, increasing shear stress could propagate the dissection [10]. Hohri et al. [11] studied three healthy aortas and three patients with Type A aortic dissection using CFD tools to investigate the WSS and OSI. They used mass flow rate waveforms as inlet boundary conditions at the ascending aorta and outlet pressure waveforms. The simulations used the turbulent (k-epsilon) transient analysis with the Newtonian blood properties assumption. The results illustrated high OSI contours at the ascending aorta or the actual entry site of the AD geometries compared to the healthy geometries of an aorta. These results are promising, but one important limitation was the difficulty in comparing different patients to each other. Takeda et al. [12] investigated eight cases of AD with both Type A and B AD, and three healthy aortas, using CFD modelling to investigate WSS and flow velocity using the k-epsilon, mass flow rate as an inlet boundary condition, and pressure waveforms at the outlet. Their results indicated that Type A tears are at a higher risk of arterial damage due to the pulsatile flow. However, one of the limitations of this study was that the authors used literature boundary conditions, which could not provide realistic results to address medical conditions such as hypertension. Wang et al. [13] used CFD modelling for a post-surgery Type A dissection in a 74-year-old male patient. The authors performed their computational analysis with the following conditions: Newtonian blood properties, solved as viscose laminar with transient analysis, using clinical boundary conditions, such as flow rate at the inlet and pressure waveform at the outlet. Their results indicated that the aorta had a higher blood flow rate in the true lumen than in the false lumen. Also, their results showed a high TAWSS, close to the tears at the false lumen; however, there were low values of TAWSS at the false lumen. This study illustrated promising outcomes; however, its patient-specific model did not contain clear information about the patient prior to developing an AD. These types of laminar and turbulent simulations were confirmed by other studies, with Type A [14] solved as a turbulent model and Type B [15] solved as a laminar model with low Reynolds numbers. Zhu et al. [14] simulated Type A aortic dissection repair for four patients. Their results showed a pressure difference between true and false lumens at different cross-sections from the descending thoracic to the abdominal aorta. The pressure difference increases with an increase in the aortic diameter during different periods, which causes aortic growth after AD repair.

Ong et al. [16] presented a systematic review using CFD tools to assess diseased aortas. Several articles studied both Type A and B aortic dissection to determine the following parameters: pressure, velocity, WSS, TAWSS, and OSI in 20 different articles. TAWSS and OSI are calculated based on the WSS in the transient analysis since it is directly proportional to the shear force of the blood flow onto the arterial wall and, specifically, the endothelial cells. For example, WSS was assessed by Osswald et al. [17] in a patient with retrograde aortic Type A dissection (RTAD). The results showed that WSS could be distal to the origin of the subclavian artery, wherein CFD was able to assess this risk.

The following points indicated the gap in the literature concerning Type A AD studies:

1. Type A is not well studied using CFD modelling due to difficulties in performing the required CT scan images, as some patients die in the community prior to hospitalisation.
2. To the best of our knowledge, few studies have investigated the utility of CFD in the same patient (i.e., before and after the aortic dissection), as this kind of study would require months to years of waiting for the development of the patient’s condition.
3. Many CFD studies focus on the pressure and velocity profiles in the true and false lumen, mainly in Type B patients and a few Type A patients, with a focus on the WSS, TAWSS, and OSI.
4. The literature did not investigate critical parameters such as relative residence time (RRT) and endothelial cell activation potential (ECAP).

Therefore, the current study presents an analysis of a patient diagnosed with a known 52.79 mm ascending aneurysm where, due to the patient’s health condition, a nonoperative approach was adopted at this diameter, and 9 months after the initial scan, the patient was diagnosed with Type A aortic dissection, which led to patient death. A CFD comparison between both scans, considering hemodynamic parameters, was performed for these data to develop the tools for predicting the severity of the disease, avoiding fatalities in similar patients and addressing the gaps in the current published literature.

## 2. Materials and Methods

### 2.1. Patient-Specific CT Scans

**Figure 1:**
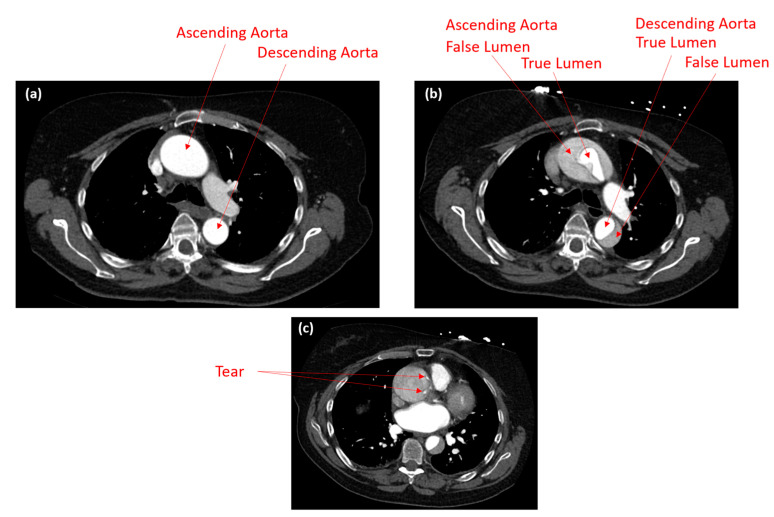
(**a**) The CT scan for the before AD event showing the ascending and descending aorta; (**b**) the CT scan showing the post-AD event when the dissection occurred at the ascending and descending aorta showing the false and true lumen; (**c**) the location of the tear for the Type A AD.

A 72-year-old female patient was diagnosed with an aneurysm at the ascending aorta. The aneurysmal diameter was below the threshold for immediate treatment, in balance of the risk of surgery and the risk of aortic complications. Scans were obtained within the Health and Disability Ethics Committees (HDECs) number: HDEC 20/NTB/217. The scans were obtained with the following values: 120 kV, 120 mAs, 241 mA, 500 ms, slice thickness 1.0 mm, and FOV 375 mm, as shown in Figure 1a. For the pre-AD event, the ascending aorta diameter was 52.79 mm, whilst the descending aorta diameter was 28.44 mm. However, for the post-AD event (as shown in Figure 1b), the ascending aorta’s true lumen had a diameter of 23.17 mm, and false lumen had a diameter of 44.55 mm, whilst the descending aorta’s true lumen diameter was 20.18 mm, and the false lumen diameter was 19.11 mm. These values are for one-slide images as the cross-sectional areas change; therefore, the 3D geometry is essential for determining the development of the dissection. Figure 1c shows the location of the tear for the post-AD event. 

### 2.2. 3D Geometry

**Figure 2:**
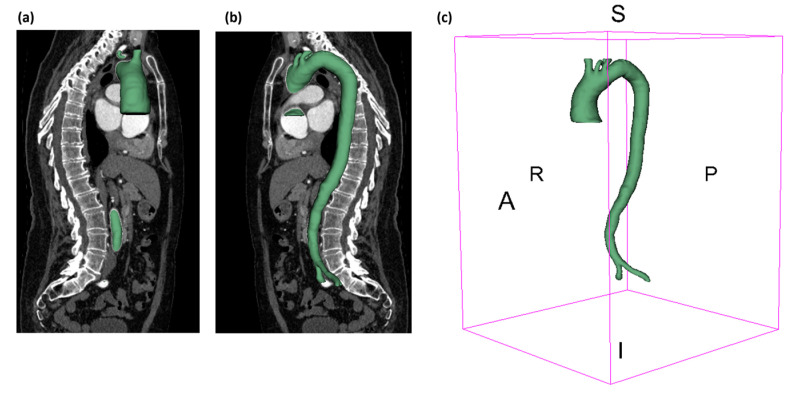

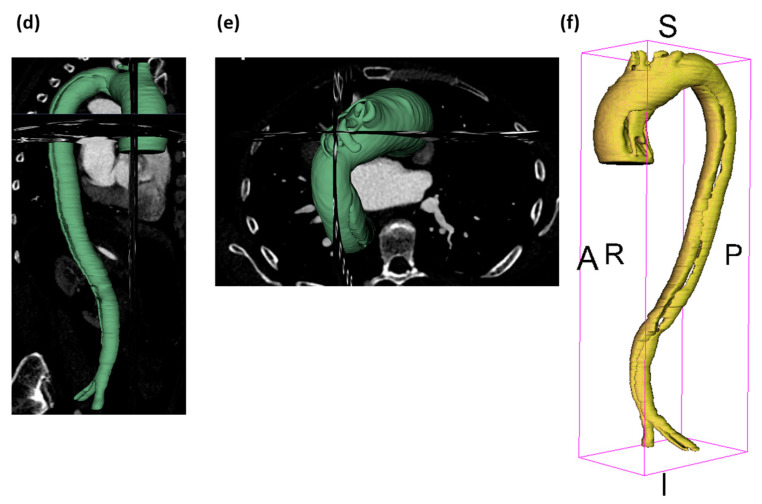
The lateral view: (**a**) back and (**b**) front for the aorta; (**c**) the ISO metric view showing the aorta geometry using 3D Slicer; (**d**) the lateral view for the post-AD event; (**e**) the top view showing the aortic branches for the post-AD event; and (**f**) the 3D model for the post-AD event when the aorta was dissected from ascending to the iliac bifurcation.

The CT scan images for both events were converted to 3D geometry using 3D Slicer (https://www.slicer.org/, open-source software accessed on 20 December 2023). Then, the 3D geometries were smoothened and converted to the mesh using Autodesk Meshmixer (www.meshmixer.com, accessed on 1 February 2024) to create the following geometries for before and after, as shown in Figure 2. Under the Meshmixer, the shape of the aorta was preserved using a smoothing filter and then clipped at the two iliac arteries and aortic branches to create the outlet boundary conditions. For the inlet boundary condition, the ascending aorta was clipped distally so that the flow was not influenced, following the process performed in our previous publications [18,19].

### 2.3. Mesh

The meshes for the before and after cases were performed using a mesh mixer and then enhanced using ANSYS fluent meshing to convert them to a polyhedral mesh. For each case, three meshes were investigated with respect to WSS_max_ and Wall Y+ for the steady case. Then, the maximum pressure and velocity at planes representing the ascending and descending aorta were investigated. For the post-AD event, we took the false and true lumens for the ascending and descending aorta. The Wall Y+ for the pre-AD event was <2, and the final mesh taken with a 1.4 value generated 949,210 elements. However, for the post-AD event, the Wall Y+ values were as follows: 2.85 for 1,408,138 elements, 2.69 for 2,270,812 elements, and 1.9 for 2,328,909 elements due to the complexity of the geometry. These analyses were performed using a processor with the following specifications: 13th Gen Intel(R) Core (TM) i7-13700F, 2.10 GHz, and 64 GB RAM. Based on that, we further investigated the models with high mesh elements: 949,210 for the before dissection and 2,270,812 for the after dissection, as shown in Table 1. 

### 2.4. CFD Setup and Boundary Conditions

**Figure 3:**
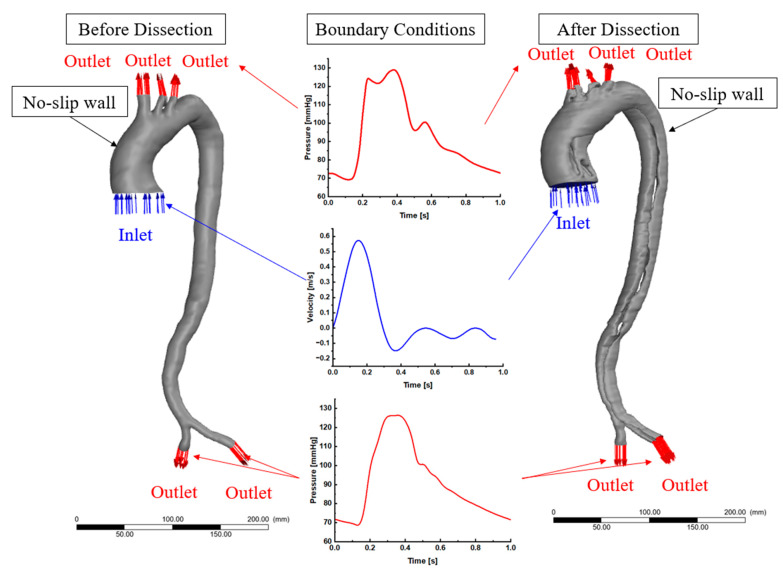
The computational domain for both pre- and post-dissection showing the boundary conditions.

In this study, the aorta models were set as non-Newtonian by assuming the blood properties with 1060 kg/m^3^ density and using Carreau–Yasuda ($\mu =\mu_{\infty }+\frac{\left(\mu_{o}-\mu_{\infty }\right)}{{\left(1+{\left(\lambda \dot{\gamma }\right)}^{2}\right)}^{\frac{1-n}{2}}}$) for turbulence model k-omega shear stress transport (SST). The following values were assumed: the infinite shear viscosity (Pa.s) $\mu_{\infty }=0.0035$; the zero shear viscosity (Pa.s), $\mu_{o}=0.056$; the time constant (s), $\dot{\gamma }=3.313$; and the power law exponent n=0.3568. The inlet boundary condition was set to inlet velocity waveforms based on the literature [19] for both before and after aortic dissection. For the outlets, we used pressure waveforms based on the literature [19]. These boundary conditions were performed using no-slip wall conditions and rigid wall assumptions, as shown in the computational domain (Figure 3). Due to the high Reynolds number for both cases, this study used turbulent flow regime analysis with viscous SST k-omega and a pressure-based solver using absolute velocity formulation. The transient analyses for each case were set to 10^−5^ time residual to achieve the convergence criteria for the continuity equations for the velocity in *x*, *y*, and *z* directions for k and omega. The computational simulations of the unsteady case for the blood flow through the aortic geometry was performed using the Navier–Stokes equations for mass conservation (1) and conservation of momentum (2).

(1) $$\rho \left(\frac{du}{dt}+u\cdot \nabla u\right)=-\nabla p++\nabla \cdot (\mu (\dot{\gamma })\nabla u)$$

(2) ∇·u=0

where u is the velocity waveform assumed in the boundary conditions as shown in Figure 3, p is the pressure waveform for the blood as shown in Figure 3, and ρ and μ are the material properties for the blood as described above. The k-ω SST equations are as follows: the transport equation for k is Equation (3), and the transport equation for ω is Equation (4).

(3) $$\frac{\partial (\rho k)}{\partial t}+\nabla \;\cdot \left(\rho Uk\right)=\nabla .\left(\left(\mu +\frac{\mu_{t}}{\sigma_{k}}\right)\nabla k\right)+P_{k}-D_{k}$$

(4) $$\frac{\partial (\rho \omega )}{\partial t}+\nabla \cdot \left(\rho U\omega \right)=\nabla \cdot \left(\left(\mu +\frac{\mu_{t}}{\sigma_{k}}\right)\nabla \omega \right)+\frac{\gamma }{v_{t}}P_{k}-{\beta \rho \omega }^{2}+2\left(1-F_{1}\right)\frac{\rho \sigma_{\omega 2}}{\omega }\nabla k:\nabla \omega$$

Note: $\nabla k:\nabla \omega =\frac{\partial k}{\partial x_{j}}\frac{\partial \omega }{\partial x_{j}}=\frac{\partial k}{\partial x}\frac{\partial \omega }{\partial x}+\frac{\partial k}{\partial y}\frac{\partial \omega }{\partial y}+\frac{\partial k}{\partial z}\frac{\partial \omega }{\partial z}$

where $\omega C_{\mu }k\;$ describe the dissipation rate of turbulent kinetic energy (k), $P_{k}$ is the production of turbulent kinetic energy, and γ is the turbulence intermittency that presents the percentage of time that turbulent fluctuations are present in the boundary layer (with a value between 0 for laminar and 1 for turbulent) to indicate the state of the flow locally. $D_{k}$ is the dissipation rate term which can be replaced by ${\;D}_{k}*\min\left(\max\left(\gamma ,0.1\right),1.0\right)\;$ when $\gamma =1=>D_{k}$ represents the Turbulent Boundary layer and γ=0, wherein $0.1D_{k}$ represents the Laminar Boundary layer. For $\mu_{t}=\frac{a_{1}\rho k}{max(a_{1}\omega ,SF_{2)}}$ (turbulent/eddy viscosity), $F_{2}=\tanh({arg}_{2}^{2})$ and ${arg}_{2}=\max\left(\frac{2\sqrt{k}}{\beta *\omega d},\frac{500v}{\omega d^{2}}\right)$, where d is the distance to the closest wall. The turbulent stress tensor is solved using the following equation:

(5) $$\tau_{ij}=-\rho U=\mu_{t}\left(\frac{\partial u_{i}}{\partial x_{j}}+\frac{\partial u_{j}}{\partial x_{i}}-\frac{2}{3}\frac{\partial u_{k}}{\partial x_{k}}\delta_{ij}\right)-\frac{2}{3}\rho k\delta_{ij}$$

### 2.5. Validation

**Figure 4:**
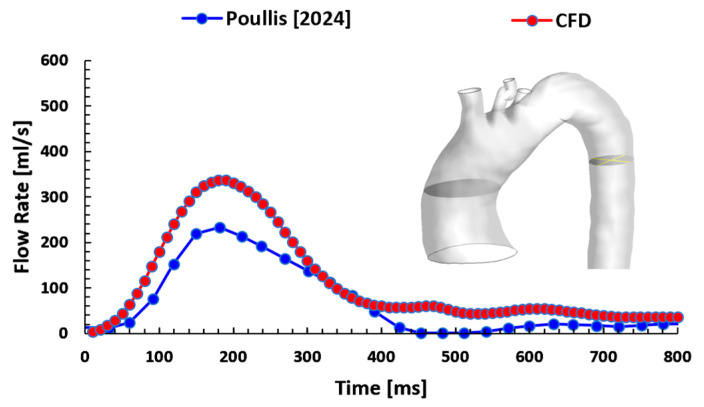
The CFD validation against the clinical data published by Poullis [5] regarding the flow rate in mL/s.

The CFD results for the pre-AD scan were validated against the MRI data published by Poullis, M. [5] regarding the flow rate in mL/s as shown in Figure 4. The current equivalent diameter for the descending aorta was 25.2 mm and converting the velocity waveform to flow rate and comparing it. The difference between these waveforms was 31% due to the systolic pressure of 120 mmHg for Poullis [5]; however, in this study, the systolic pressure was 140 mmHg, as this patient had an aneurysm in the ascending aorta. Given this, the equivalent diameters for both cases are very close, and the flow rate trend is very similar. The current results indicate that the CFD boundary conditions develop an acceptable trend for the waveform profiles along the aorta. 

## 3. Results

### 3.1. Blood Pressure and Velocity Profiles 

**Figure 5:**
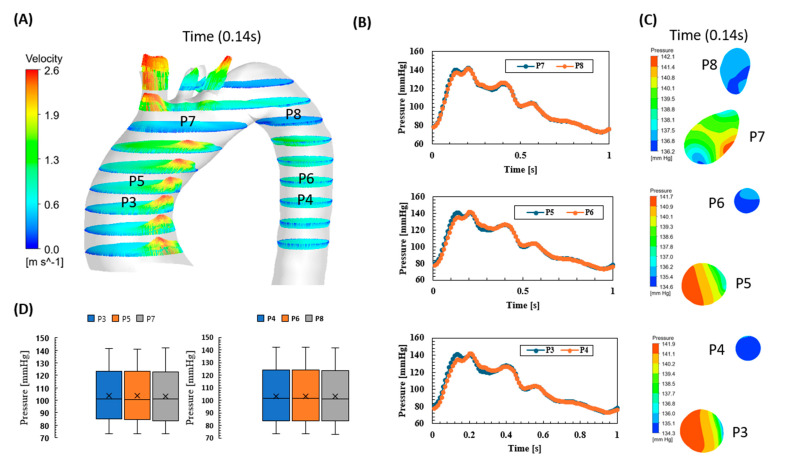
Pre-aortic dissection: (**A**) the velocity vector for the ascending aorta, descending aorta, and aortic arch with the bifurcation, at the systolic (0.14 s); (**B**) the pressure waveforms at the three selected planes to compare between ascending and descending pressure profiles; (**C**) the pressure contours at the systolic time (0.14 s); and (**D**) the maximum, average, and minimum pressure at the selected points shown in (**A**).

Blood pressure waveforms reflect the progression of the disease and hence will be examined in terms of how they reflect the progress from the initial aneurysm at the ascending aorta to an aortic dissection. In this study, the pressure waveforms for one second were obtained from ANSYS Fluent at different planes proximal and distal to the dissection. Figure 5 shows the pressure waveforms at the ascending and descending aorta and close to the aortic arch. At the same time, Figure 5 shows the velocity vectors at different planes on the aorta, from the ascending aorta, the aortic arch and the supra-aortic branches, and the descending aorta. Figure 5A shows the velocity vector at the systolic (peak time 0.14 s), which indicates a high vector profile developed at the ascending aorta and the bifurcations of the supra-aortic branches. These high vector velocities reached a maximum value of 2.6 m/s, which could present a high risk to the internal lumen intimal layer. This is further investigated in the next section on wall shear stress (WSS). Figure 5B shows the pressure waveform difference at the same plane but for the ascending and descending aorta, whilst we simultaneously took contour profiles for the pressure at the peak time of 0.14 s to explore the maximum pressure value with 142.1 mmHg. Figure 5D compares the pressure minimum, average, and maximum values for the points at the ascending and descending aorta sections. The results indicate a trend for the pressure waveforms to be acting in a similar fashion for these planes; however, there was a clear diagnosis that the patient was under an ascending aneurysm situation. Based on the decisive element in diagnosing, the increase in the ascending diameter can be observed from the CT scans, as shown in Figure 1a [20]. 

**Figure 6:**
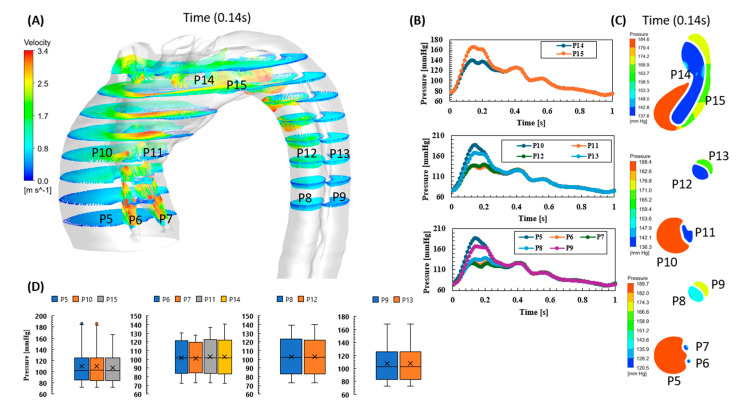
Post-aortic dissection: (**A**) the velocity vector for the ascending aorta, descending aorta, and aortic arch with the bifurcation at the systolic (0.14 s); (**B**) the pressure waveforms at the three selected planes to compare between ascending and descending pressure profile; (**C**) the pressure contours at the systolic time (0.14 s); and (**D**) the maximum, average, and minimum pressure at the selected points shown in (**A**).

**Figure 7:**
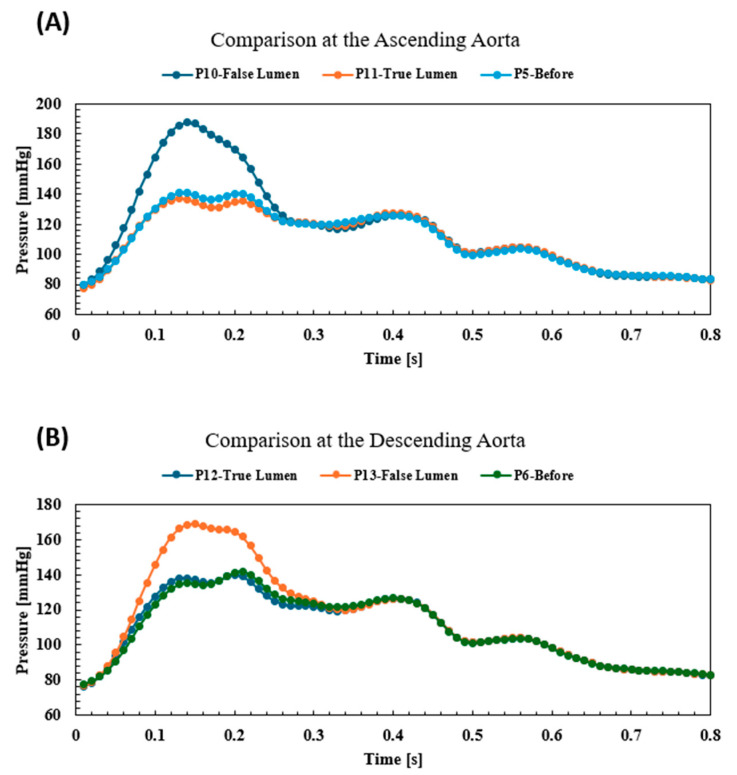
(**A**) The pressure waveforms at the ascending aorta for both before and after the aortic dissection, and (**B**) at the descending aorta.

Figure 6 shows the pressure and velocity profiles for the aortic dissection. Figure 6A shows the velocity vectors at the peak (0.14 s) and how the high velocity reached up to 3.4 m/s, compared to a high velocity which reached a high of only 2.7 m/s in the before-AD event. Also, the high velocity vectors reached at the descending aorta were 0.74 m/s for the true lumen and 1.02 m/s for the false lumen compared to 0.87 m/s in the before-AD event. The same situation can be seen for the pressure waveforms, which show high elevations at the false lumen, at both the ascending and descending aortas. This result was clear at both planes with the data points (P5, P9, P10, and P13), as shown in Figure 6B. Figure 6C shows the pressure contours at the peak (0.14 s), which shows the peak value reached at 189.7 mmHg compared to 142.1 mmHg before the dissection event. Figure 6D shows the pressure maximum, average, and minimum values at different planes at the aorta (ascending aorta, descending aorta, and aortic arch) which indicate that the pressure values for the pre-AD event were close to the pressure in the true lumen of the dissected aorta. Figure 7 shows the comparison of pressure waveforms at the ascending and descending aorta between the true and false lumens. The results indicate that the before-AD event pressure waveform was nearly identical to the true lumen at both the ascending and descending aorta; however, the false lumen pressure waveforms showed an amplification to the systolic pressure. 

### 3.2. WSS Parameters

**Figure 8:**
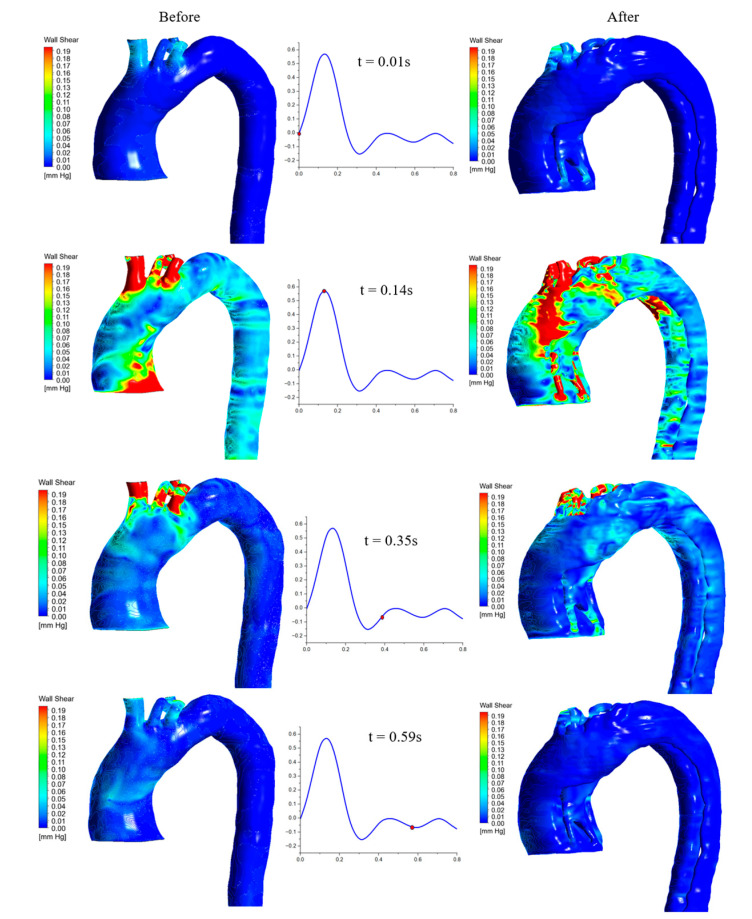
The WSS for both before and after the aortic dissection at different times, based on the inlet velocity waveform.

Wall shear stress is one of the most common parameters to assess cardiovascular diseases. The results obtained from the ANSYS Fluent were post-processed, and further calculations were performed using MATLAB. Figure 8 shows the WSS contours before and after dissection at different times during the velocity waveform: 0.01 s, 0.14 s, 0.35 s, and 0.59 s. At 0.14 s, we found the high contours for WSS in the area where the ascending aorta developed the false and true lumens after dissection. 

WSS contours are recognised with high or low readings, which could be an indicator for the cause of intimal dysfunction that leads to cardiovascular diseases such as atherosclerosis, which could then lead to aneurysms. So, it is very common to see low WSS contours with the development of ascending aneurysm due to the locally altered hemodynamics and arterial wall parameter with values ranging between 2 and 6 Pa (0.015–0.045 mmHg) and very high contours at the inlet based on the inlet boundary condition [21]. These contours indicate the development of abnormal wall dilatation in the aortic wall, which impacts its elasticity. Also, based on the clinical data, when the aortic diameter exceeds 40 mm, the size of the aneurysm size correlates directly with the development of rupture and dissection [22]. These could vary between different patients and depend on their individual risk assessment, and the severity of the aneurysm corresponds with age, body weight, and body height [23]. 

**Figure 9:**
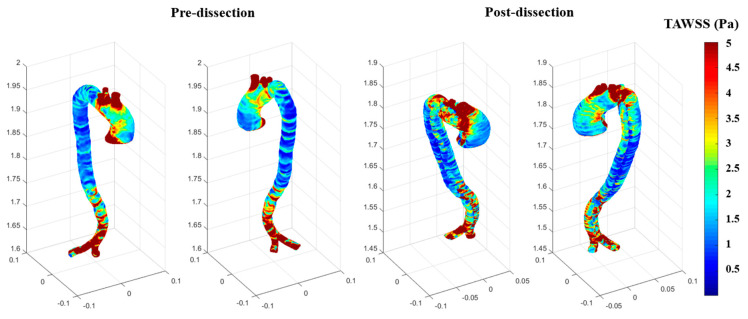
The TAWSS contours for both the pre-and post-dissections.

The results for the WSS in x, y, and z and their coordinates were further explored to calculate the time-average wall shear stress (TAWSS), as shown in Figure 9. The TAWSS illustrates the average tangential forces exerted by the blood flow used as an inlet boundary condition on the endothelial surface of the aortic wall over a cardiac cycle [24,25]. The comparison between the before and after in terms of TAWSS shows that the reduction of TAWSS around the ascending aorta during aneurysm development contributes to endothelial dysfunction, which results in an increase in high contours of TAWSS at locations where aortic dissection could occur, such as in the post-AD event. 

The TAWSS is used to calculate the oscillation shear index (OSI), which is based on using the WSS in the three-dimensional coordinates to address the blood flow and the endothelial cells’ behaviour on the arterial wall surface using the following equation: OSI = $\;\frac{1}{2}\left(1-\frac{\left|\int_{0}^{T}WSS\;dt\right|}{TAWSS}\right)$, with the focus on the flow oscillation during a cardiac cycle (T). 

**Figure 10:**
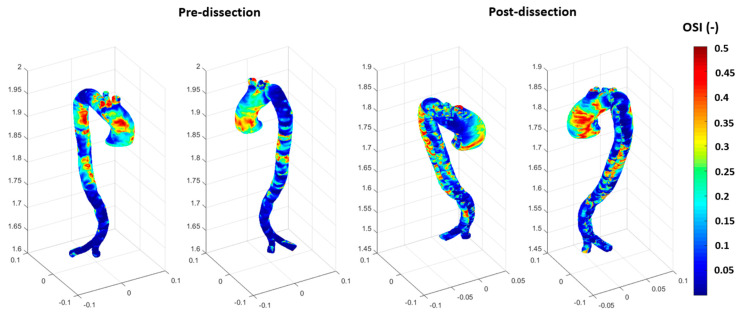
The OSI for both the pre-and post-dissections.

OSI is useful for studying the directional changes in WSS over a cardiac cycle during abnormalities to the arterial wall. It helps track the development of cardiovascular diseases at the ascending aorta. The range of the dimensional OSI is from 0, meaning that the WSS vector is in one direction, and 0.5, which means the vectors are equally oscillatory in their directions, which presents a high level of oscillation. Due to the complex curvature of the aortic geometry, low OSI values indicate that the blood flow is stable and unidirectional, which will cause less harm to atherogenesis. Comparing the TAWSS and OSI (Figure 9 and Figure 10) shows that the TAWSS were more illustrative of the high contours, with a maximum of 5 Pa (0.04 mmHg) at the end of the aorta for the before model, which presents the outlet’s boundary conditions. However, the TAWSS contours reached the maximum value of 10 Pa for the after-dissection model at the aortic arch and the bifurcation of the supra-aortic branches. These differences could be due to the low oscillation WSS occurring at the aortic aneurysm, as seen in Figure 8, when at the maximum blood flow. This usually results in the development of endothelial dysfunction and inflammation, which restructures part of the arterial wall and is part of developing a severe aneurysm. Therefore, the ascending aneurysm in the pre-dissection model causes a weakness in the aortic wall (including the ascending aortic arch and the descending aorta), which resulted in the dissection, as shown in Figure 8 for the WSS at 0.14 s, Figure 9 for the TAWSS at the aortic arch, and Figure 10 for the OSI. The development of the ascending aortic wall tear was due to the rupture of the ascending aneurysm.

**Figure 11:**
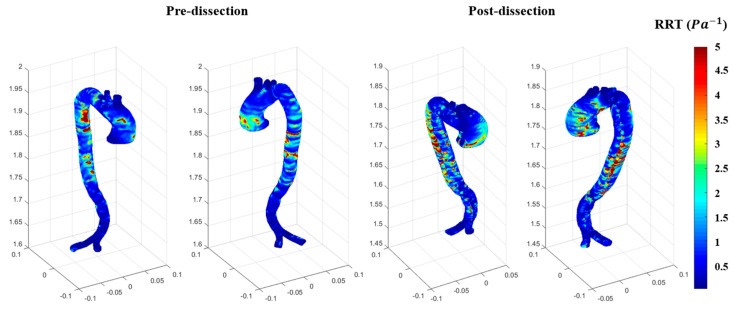
The RRT is for both the pre-and post-dissections.

The OSI results illustrate the directional changes in blood velocity waveforms over a cardiac cycle. Figure 10 shows an increase in the OSI contours at the ascending aorta in the before-case due to the blood flow distribution, as its regime changed from laminar to turbulent, which is managed by the k-omega SST mode. For the after-dissection model, the OSI remained high due to the oscillatory flow (turbulent regime), generated by high regions of hemodynamic stresses, such as WSS at the inner layer of the arterial wall. Therefore, the after-dissection event occurred due to the pathogenesis of aneurysmal development or other vascular conditions such as genetic predisposition. Another hemodynamic parameter used for localising endothelial dysfunction and inflammation, which could lead to cardiovascular diseases, is the relative residence time (RRT). RRT is proportional to the residence time of the blood particles near the arterial wall, calculated based on the relationship between the OSI and TAWSS $\left(RRT=\frac{1}{\left(1-2*OSI\right)TAWSS}\right)$. Figure 11 shows the RRT for both before and after dissection, and it can be observed that high RRT has developed at the ascending aorta for the before dissection event. The RRT presents the time of blood waveform travel inside the aorta during a cardiac cycle. High contours of RRT are associated with regions that could cause aneurysm formation, as seen in Figure 11 for the before-dissection event. The results of RRT, as shown in Figure 11, show one side of the ascending aorta with low contours of RRT and the other side a spot of high contours of RRT. These spots of high contours increased in the after-dissection event at both sides of the ascending aorta. 

**Figure 12:**
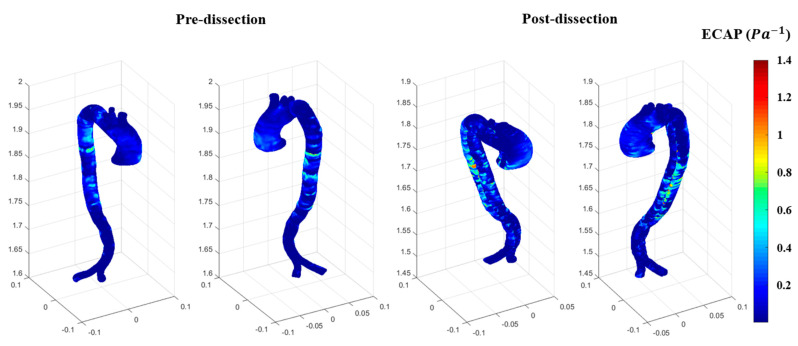
The ECAP for both the pre-and post-dissections.

The results of the RRT indicate that the ascending aorta was subject to high shear stresses in the pre-AD event compared to post AD event due to the average time of the blood flow circulate at the ascending aorta; however, post-dissection, the cross-sectional area became the false and true lumens. To the best of our knowledge, the RRT has not been investigated for this type of aortic dissection, which shows high contours of RRT spots present at the ascending and descending aorta. RRT could be used as a potential tool for identifying the slowing down of blood flow accompanied by low shear stress, which could impact the compliance of the arterial wall. If this type of impact is ongoing due to the development of an aneurysm, it could lead to endothelial cell activation, which could cause inflammation. Therefore, it is worth investigating the endothelial cell activation potential (ECAP) ratio between the OSI and the TAWSS. ECAP is a useful hemodynamic parameter to assess the endothelial cells whose intraluminal surface is subject to blood flow abnormality due to cardiovascular disease development. Figure 12 shows the ECAP for both before and after dissection, in which high contours are present at the same locations for the before and after dissection events. This is due to the use of non-Newtonian blood properties, which could address the high ECAP regions and lead to thrombosis. 

## 4. Discussion

This study demonstrates the feasibility of utilising computational fluid dynamics in the prediction of potential sites of aortic dissection occurring in the future. The novelty of this study rests on the fact that we have access to pre-dissection CT imaging and that we are able to compare this to the index CT imaging at the time of dissection for the same patient. From this, we were able to utilise available computational fluid models to accurately predict the entry tear site. 

Various studies have been published looking into the application of computational fluid dynamics in aortic dissection. A study by Alimohammadi et al. demonstrated the application of CFD by simulation using Windkessel boundary conditions and with the assumption that the arterial wall is a rigid model. By assuming blood properties as non-Newtonian (Carreau–Yasuda) using the k-omega shear stress transport (SST) model, they were able to demonstrate the high wall shear stress (WSS) contours in the regions of interest in aortic dissection. This variable is significant in preventing further propagation of the dissection and avoiding unwanted complications [9]. In contrast, Hohri et al. looked into the assumption of blood properties as Newtonian, and applying the turbulent (k-epsilon) transient analysis, they were able to demonstrate high oscillating shear index (OSI) contours at the ascending aorta or the actual entry side of the aortic dissection compared to a normal healthy aorta [11]. 

The application of computational fluid dynamic models has demonstrated how pulsatile flow itself is detrimental and results in further propagation in Type A aortic dissection [12]. High values of time-averaged wall shear stress (TAWSS) have also been demonstrated close to the tears at the false lumen with contrasting low values of TAWSS seen at the false lumen. This can be applied into clinical practice in predicting false lumen thrombosis or even future aneurysmal dilatation and the need for surgical intervention [13,14]. With the utilisation of various equations to measure WSS, OSI, and TAWSS, there remains a scarcity of studies looking into the utilisation of relative residence time (RRT) and endothelial cell activation potential (ECAP) as part of the CFD modelling. In our study, we demonstrated how RRT itself provides valuable data in predicting aneurysmal degeneration or the potential site of aortic dissection, and ECAP will be able to predict false lumen thrombosis. 

Our study is limited by a small sample size, and hence further studies are warranted to evaluate this model with a larger sample population. The application of real-time patient-specific haemodynamic data are missing due to specific assumptions as part of the modelling equation. The availability of this information is crucial to be able to further strengthen the predictive accuracy of this model to hopefully be applied in future clinical practice and guide clinicians with ongoing management. Our current study is limited to the assessment of pre- and index dissection imaging only. The utility of computational fluid dynamics in predicting the prognosis of false lumen thrombosis and aneurysmal dilatation of the aorta was thus not explored and would be a topic of future ongoing research by utilising same patient follow-up imaging data. 

Future research work in the utilisation of CFD in aortic dissection would aim to apply patient-level haemodynamic data to evaluate the current model and assumptions in the CFD analysis. Following this, the data can then be utilised further in ongoing patient follow-up to predict disease progression: aneurysmal formation, false lumen thrombosis, or dissection propagation. Information from this is crucial in guiding clinicians in educating patients and deciding the timing or plan for intervention if needed. CFD analysis can be expanded further to develop a predictive model that can be employed in patients with a genetic predisposition to aortic dissection, as their underlying pathophysiology for the development of aortic dissection might be slightly different and potentially more rapid in comparison to other patients. In terms of future healthcare resource allocation, CFD analysis can be harnessed to distinguish patients that require close surveillance compared to those that do not, in terms of predicting false lumen thrombosis. 

## 5. Conclusions

In conclusion, the model correctly detected an aneurysm at the ascending aorta in the before AD event and provided indicators for a potential AD development; however, we cannot predict the timing of the occurring AD complication, nor whether surgical intervention would have altered the final outcome for the patient. The computational results indicated high WSS contours at the ascending aorta, which exceeded the normal range and could have influenced the development of the dissection. In this case, the patient was subject to a severe Type A AD, and this was for the first time investigated computationally based on CT scan data from before and after the dissection event. The pressure waveform results for the before-dissection event, when the patient was only subjected to an ascending aortic aneurysm based on the first CT scan, indicated similar trends for these waveforms at the ascending and descending aorta, which exceeded those for a healthy case. For the after-dissection CT scan data, these pressure waveforms showed different trends, such as the high systolic pressure for both false lumens at the ascending and descending aorta compared to the true lumen with different amplitudes. This outcome was observed at the different planes of the ascending and descending aorta, and the same outcome was observed at the aortic arch as well. Also, the OSI contours showed an elevation at the time of ascending aneurysm (before dissection), and then the elevation dropped at the location of the tear, causing the dissection. These outcomes warrant further investigation using the patient clinical boundary conditions for further statistical analysis to develop a map for the progression of this disease. 

Further verification of the current results is required, particularly through replication of this analysis with a larger sample size of patients. Additionally, acquiring follow-up CT scans will be crucial in developing a clinical tool for the effective management of patients presenting with AD. Future research should also focus on exploring the utility of CFD in predicting the prognosis of false lumen thrombosis and aneurysmal dilatation of the aorta based on structural and hemodynamic parameters.

Once validated with a larger cohort of patients and comprehensive follow-up data, the developed approach and computational framework have the potential to assist clinicians and contribute to the early detection of AD evolution and the prevention of fatal outcomes, such as wall rupture.

## Figures and Tables

**Table 1:** The mesh test data for the pre-dissection and post-dissection showing the maximum WSS, maximum pressure, and Wall Y+.

Mesh #	Cells	WSS_max_ [Pa] Steady Case	Pressure_max_ [mmHg] Ascending/Descending	Velocity_max_ [m/s] Ascending/Descending	Wall Y+ for Steady Case
Before-1	412,352	9.1	139.58/141.43	0.63/0.87	1.91
Before-2	414,748	11	140.48/141.86	0.63/0.87	1.77
Before-3	949,210	11	141.66/141.83	0.63/0.87	1.41
After-3	1,408,138	12	186.144/135.06 136.42/167.58	0.63/0.59 0.97/0.77	2.85
After-2	2,270,812	13.92	186/136.2 137.08/167.36	0.63/0.81 0.912/0.766	2.69
After-1	2,328,909	17.98	188.08/136.98 139.80/168.59	0.64/0.44 1.01/0.74	1.9

## Data Availability

Due to patient privacy, the datasets generated and analysed during the study are not publicly available.
